# Nausea, Vomiting, Abdominal Pain, and Acute Kidney Injury in a Kidney Transplant Recipient

**DOI:** 10.34067/KID.0000001038

**Published:** 2026-04-30

**Authors:** Abinay Siva Kumar Reddy Vanteru, Krishna Vani Nemalidinne, Venkata Manchala

**Affiliations:** University of Arkansas for Medical Sciences, Little Rock, Arkansas

**Keywords:** AKI, acute rejection, aldosterone, chronic hemodialysis, CKD, dialysis, glomerular disease, hyponatremia, peritoneal dialysis, transplantation

## Abstract

**Figure:**
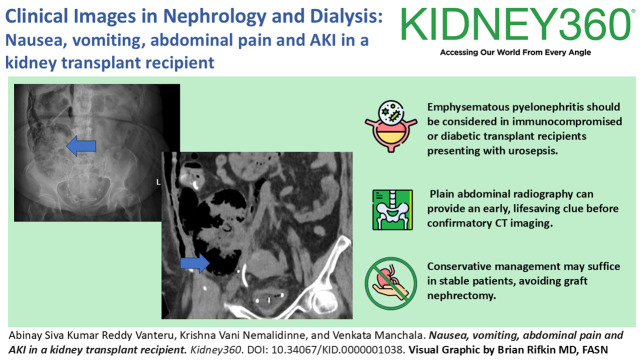

## Case Description

A 62-year-old woman presented with abdominal pain, nausea, vomiting, and hematuria 1 month after a deceased donor kidney transplant. She had received alemtuzumab and high-dose steroids for induction and was on tacrolimus, mycophenolate, and prednisone for maintenance immunosuppression.

On arrival, her BP was 90/60 mm Hg and heart rate was 95 bpm. Examination revealed tenderness over the right lower quadrant. Laboratory tests showed glucose 581 mg/dl, creatinine 10.1 mg/dl (baseline 2.6 mg/dl), potassium 8.1 mmol/L, bicarbonate 9 mmol/L, leukocytes 20,000/*µ*l, and elevated *β*-hydroxybutyrate, consistent with diabetic ketoacidosis (DKA) and AKI. Urinalysis revealed hematuria, pyuria, glucosuria, ketonuria, and bacteriuria.

**Figure 1:**
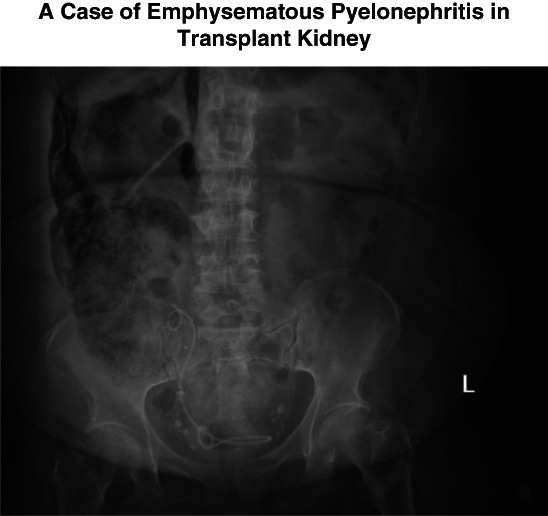
X-ray of the abdomen erect at initial presentation demonstrates significant amount of retroperitoneal free air seen along the right flank and right paraspinal region and diffuse emphysematous changes involving the right transplant kidney.

**Figure 2:**
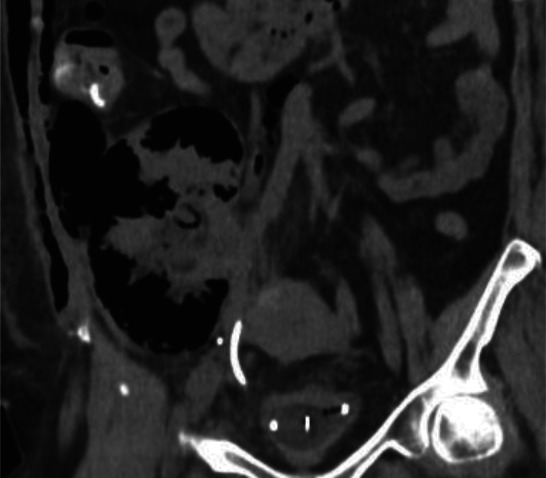
**CT of the abdomen without contrast on initial presentation demonstrates emphysematous pyelonephritis within the transplanted kidney with intraperitoneal, right retroperitoneal free air, and subcutaneous emphysema along the right abdominal wall.** CT, computed tomography.

An abdominal radiograph demonstrated retroperitoneal free air outlining the renal allograft (Figure 1). She was treated for DKA and urosepsis with intravenous fluids, insulin, and broad-spectrum antibiotics. Persistent hyperkalemia required emergent hemodialysis. Contrast-enhanced computed tomography (CT) performed 24 hours later confirmed emphysematous pyelonephritis (EPN) involving the transplant kidney, with extensive parenchymal necrosis, perinephric collection (4.5×3.8 cm), and air tracking into the retroperitoneum, abdominal wall, and mesentery (Figure 2). Blood and urine cultures grew *Escherichia coli*.

**Figure 3:**
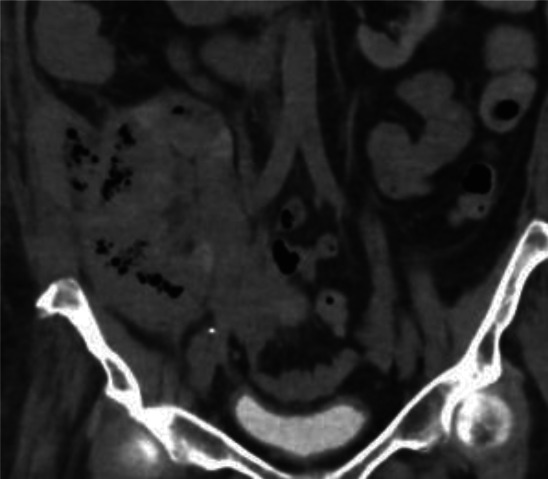
**CT of the abdomen without contrast 1 month later demonstrates interval improved foci of air within the transplant kidney.** There is perinephric fluid collection concerning for abscess.

Tacrolimus and mycophenolate were held, while low-dose prednisone (5 mg daily) was continued. The patient improved clinically on medical therapy alone; nephrectomy was deferred. The perinephric collection spontaneously drained through the inferior aspect of the Gibson incision. At the 3-month follow-up, serum creatinine stabilized at 4 mg/dl, and repeat CT showed near-complete resolution of gas and perinephric collection (Figure 3).

## Discussion

EPN is a rare, life-threatening necrotizing infection of the renal parenchyma caused by gas-forming bacteria, most commonly *E. coli* or *Klebsiella* species. In renal transplant recipients, diabetes and immunosuppression are key predisposing factors.^1^ Because grafts are denervated, flank pain may be absent, delaying diagnosis. Although CT is the diagnostic gold standard, plain abdominal radiography may be sufficient to recognize intraparenchymal gas and prompt early management.

Historically, nephrectomy was the standard of care; however, recent experience supports conservative management in selected hemodynamically stable patients who respond to antibiotics and supportive therapy. Temporary withdrawal of immunosuppression, glycemic control, and drainage of perinephric collections is essential.^2^ Graft salvage is achievable in many cases without surgery.

## Teaching Points

- Emphysematous pyelonephritis should be considered in immunocompromised or diabetic transplant recipients presenting with urosepsis.
- Plain abdominal radiography can provide an early, lifesaving clue before confirmatory CT imaging.
- Conservative management may suffice in stable patients, avoiding graft nephrectomy.
